# Trends and factors associated with delivery hospitalizations involving severe maternal morbidity in portuguese public hospitals: A population-based study (2010–2018)

**DOI:** 10.1038/s41598-026-42363-9

**Published:** 2026-04-04

**Authors:** Catarina Camarinha, Maria Miguel Oliveira, Miguel de Araújo Nobre, Cristina Furtado, Cecília Elias, Leonor Bacelar-Nicolau, Andreia Silva Costa, Paulo Jorge Nogueira

**Affiliations:** 1 Faculdade de Medicina, EPI Task-Force FMUL, Universidade de Lisboa, Lisbon, Portugal; 2 Área Disciplinar Autónoma de Bioestatística (Laboratório de Biomatemática), Faculdade de Medicina, Universidade de Lisboa, Lisboa, Portugal; 3 Faculdade de Medicina, Clínica Universitária de Estomatologia, Universidade de Lisboa, Lisboa, Portugal; 4 Faculdade de Medicina, Instituto de Saúde Ambiental, Universidade de Lisboa, Lisboa, Portugal; 5 Faculdade de Medicina, Instituto de Medicina Preventiva e Saúde Pública, Universidade de Lisboa, Lisboa, Portugal; 6 Unidade de Epidemiologia, Faculdade de Medicina, Instituto de Medicina Preventiva e Saúde Pública, Universidade de Lisboa, Lisboa, Portugal; 7 Instituto Nacional de Saúde Doutor Ricardo Jorge, Lisboa, Portugal; 8 Direção Geral de Saúde, Divisão de Saúde Sexual Reprodutiva Infantil e Juvenil, Lisboa, Portugal; 9 Católica Medical School, Center for Interdisciplinary Research in Health (CIIS), Universidade Católica Portuguesa, Rio de Mouro, Portugal; 10 Inovação e Desenvolvimento em Enfermagem de Lisboa, CIDNUR - Centro de Investigação, Escola Superior de Enfermagem de Lisboa, Lisboa, Portugal; 11 CRC-W-Católica Research Centre for Psychological, Family and Social Wellbeing, Universidade Católica Portuguesa, Lisbon, Portugal

**Keywords:** Severe maternal morbidity, Maternal health, Delivery hospitalizations, Diseases, Health care, Medical research, Risk factors

## Abstract

This study aimed to estimate Severe Maternal Morbidity (SMM) rates and trends among public hospital deliveries in Portugal, 2010–2018, and identify associated factors. A register-based observational cross-sectional study was conducted using public hospital admission delivery episodes. SMM was identified using the Centers for Disease Control and Prevention (CDC) indicators. Descriptive statistics, Mann–Kendall trend test, binary logistic regression and adjusted logistic regression were used to assess trends and associated factors. Between 2010 and 2018, 8,854 delivery episodes met the criteria for SMM. Blood transfusion, acute renal failure, and shock showed the highest increase in rate per 1,000 hospitalized deliveries. SMM rate was 13.14 per 1,000 deliveries and 3.22 per 1,000 deliveries without blood transfusion. Maternal age (≥ 40 years adjusted odds ratio (OR) 1.38, 95% confidence intervals (CI) 1.09–1.66), hospitalized days (adjusted OR 1.04, 95% CI: 1.04–1.05), number of diagnoses (adjusted OR 1.27, 95% CI 1.25–1.28) and procedures during hospitalization (adjusted OR 1.26, 95% CI 1.24–1.27), and International Classification of Diseases, 10th edition, Clinical Modification and Procedure Coding System (ICD-10-CM/PCS) (adjusted OR 1.63, 95% CI: 1.08–2.18) were significantly associated with a higher risk of SMM. After 2015, the risk of SMM decreased. SMM with blood transfusion was positively associated with hospitalization days, number of diagnoses, procedures, residing in Algarve, Alentejo or Centro, and ICD-10-CM/PCS. Trends in SMM between 2010 and 2018 in Portugal show an increase, particularly for blood transfusion. Risk factors included age, hospitalization length, intensity of diagnoses and procedures, and ICD-10-CM/PCS coding.

**Supplementary Information:**

The online version contains supplementary material available at 10.1038/s41598-026-42363-9.

## Introduction

Vital statistics reports on maternal mortality have been used globally as a key indicator to monitor maternal health^1,2^. According to the World Health Organization (WHO), the maternal mortality ratio in Portugal was estimated at 15 deaths per 100,000 live births between 2000 and 2023, with the lifetime risk of maternal death remaining stable^2^. The country has experienced a low sustained fertility rate, with 1.40 births per woman in 2024^3^, and a continued trend toward delayed childbearing, with the mean age at first birth reaching 30.7 years in 2024^4^. Given the overall improvements in the quality of maternal health care and low maternal mortality rates in high-income countries^2^, Severe Maternal Morbidity (SMM) surveillance has been used to monitor maternal health and quality of maternal care^5^.

SMM refers to outcomes occurring during pregnancy, labor, or the postpartum period that could have a significant impact on women’s health, either in the short or long term^5^. The SMM includes pregnancy complications such as eclampsia and amniotic fluid embolism, cardiorespiratory complications, as well as necessary interventions such as blood transfusion, hysterectomy, respiratory support, and blood transfusion^6^. These conditions can be life-threatening or cause long-term health problems, resulting in prolonged hospitalization and increased healthcare costs^7,8^.

To our knowledge, SMM evaluated at the national level in Portugal remains unknown, and factors related to SMM at the national level are not clearly defined. Collecting information on SMM in deliveries that have occurred in Portuguese public hospitals over a number of years can provide important information for obstetric care, identifying trends and possible unmet needs. The purpose of this study was to estimate the SMM rates and trends among public hospital admissions for delivery between 2010 and 2018 in Portugal and to identify the factors associated with SMM.

## Methods

### Study design and data source

A register-base observational cross-sectional study was conducted, using all public hospital admission episodes with a diagnosis related to delivery in Portugal, between 2010 and 2018. The STROBE reporting guidelines were employed for this study^9^. This study utilized anonymized secondary health data provided by the Central Administration of the Health System of the Portuguese Ministry of Health (Administração Central do Sistema de Saúde — (ACSS), under a formal data-sharing agreement with the Faculty of Medicine, University of Lisbon (FMUL), available at https://www.medicina.ulisboa.pt/sites/default/files/parcerias/2020-05/administracao-central-do-sistema-de-saude.pdf. All data were anonymized by ACSS prior to researcher access, and the authors had no access to identifiable personal information. The study did not involve any experimental procedures, interventions, or direct contact with human participants. As the research was based exclusively on fully anonymized secondary data collected for administrative and healthcare purposes, informed consent from individual subjects was not required. All data processing and analyses were conducted in full compliance with applicable national legislation and European data protection standards, including the General Data Protection Regulation. All methods were carried out in accordance with relevant guidelines and regulations.

Portugal’s healthcare system is mainly funded by taxes and offers universal coverage through the National Health Service, alongside private providers in a mixed healthcare model^10^. Data from the administrative National Hospital Discharge Database was used for this study, which includes information from Portuguese public hospitals, representing around 70% of all inpatient stays^11,12]^ and 80.4% of all deliveries^13^. Within the database, inpatient diagnoses and procedures were coded by trained medical staff according to the International Classification of Diseases, Ninth Revision, Clinical Modification (ICD-9-CM) before 2017 and with the International Classification of Diseases, 10th edition, Clinical Modification (ICD-10-CM) and procedure coding system (ICD-10-PCS) after 2017^14,15^.

### Study population

Patients were included in the study if they were recorded as an inpatient hospitalization with a primary or secondary diagnosis of delivery during the study period. Hospitalization for delivery was classified according to a method previously published by Kuklina et al^16^., which includes diagnosis codes, diagnosis-related group codes, and procedure codes to identify hospitalizations due to delivery (Supplementary Table S1). Episodes were further restricted to delivery hospital admissions for female individuals between the ages of 15 and 49 years.

### Indicators of SMM

Information indicative of SMM was identified using the Centers for Disease Control and Prevention (CDC) indicator list^6^. This list was adapted to identify maternal morbidity using hospital discharge data and contains 21 indicators with corresponding diagnosis and procedure codes (Supplementary Table S2).

### Statistical analysis

Descriptive statistics were used to assess the trends of SMM within hospital delivery episodes across the study period. The frequencies of SMM and SMM indicators were expressed by number of events and as rates (events per 1,000 deliveries), using delivery hospitalizations recorded in the discharge database as denominator, with 95% confidence intervals (CIs). SMM overall rates were also provided without blood transfusion indicator, as recommended by CDC^6^. Mann-Kendall trend tests were applied to investigate whether rates changed over time. In addition, SMM rates excluding blood transfusion were calculated by age group (< 20, 20–24, 25–29, 30–34, 35–39, and ≥ 40 years) and by Nomenclature of Territorial Units for Statistics (NUTS) level II, which includes the statistical regions of Lisbon Metropolitan Area, the region containing the national capital city of Lisbon, as well as Norte, Centro, Alentejo, Algarve, and the autonomous regions of Portugal, Madeira, and the Azores.

To identify factors associated with SMM, an analysis using binary logistic regression to estimate unadjusted odds ratios (OR) with 95% CIs was conducted. The dependent variable was defined as a binary variable for SMM. Each delivery hospitalization episode was classified as ‘1’ if at least one SMM indicator was present, and ‘0’ otherwise. Variables with *p* < 0.20 and those of theoretical relevance were included in the adjusted logistic regression model. Variables used in the adjusted logistic regression were: age group, hospitalization days, number of diagnosis and procedure recorder during hospitalization, NUTS level II, year of admission and admission type, and ICD version (ICD-9/10-CM/PCS). The area under the curve (AUC) was used to evaluate the discriminative ability of the logistic regression models. An additional sensitivity analysis was performed to evaluate the robustness of the findings using mixed-effects logistic regression models with hospital-level random intercepts.

The analyses were performed with R version 4.4.1 (R Foundation for Statistical Computing, Vienna, Austria) and with Excel (Microsoft, USA).

## Results

**Fig. 1:**
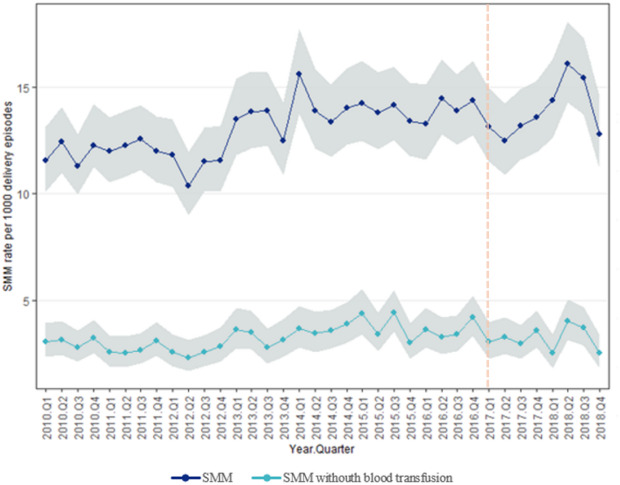
Severe Maternal Morbidity (SMM) rates per 1,000 Hospital Delivery Episodes, from 2010 to 2018, overall (dark blue line) and without the blood transfusion indicator (light blue line). The vertical dashed line indicates the transition from International Classification of Diseases, Ninth Revision, Clinical Modification (ICD-9-CM) to the International Classification of Diseases, 10th edition, Clinical Modification (ICD-10-CM) and procedure coding system (ICD-10-PCS).

A total of 673,978 delivery related hospital discharge episodes were identified in Portuguese public hospitals between 2010 and 2018. Among these 8,854 episodes were identified with at least 1 indicator that met the SMM criteria and a total of 10,278 SMM-related events during delivery and/or postpartum hospitalizations. Table 1 shows the number of SMM rates (events per 1,000 deliveries) by year (2010–2018). Figure 1 shows SMM rates with and without blood transfusion over the study period.

Between 2010 and 2018, there were 13.14 (95% CI: 12.86–13.41) episodes of SMM per 1,000 deliveries and 3.22 (95% CI: 3.08–3.36) episodes of SMM without blood transfusion indicator per 1,000 deliveries. The highest overall SMM rate was 14.70 (95% CI: 13.82–15.62) in the year 2018, and the lowest reported SMM rate was 11.33 (95% CI: 10.59–12.11) in the year 2012. During the study period, the most frequent indicator for being classified as having SMM was blood transfusion (*n* = 7,411, 72.11%), followed by disseminated intravascular coagulation (*n* = 610, 5.94%) and hysterectomy (*n* = 387, 3.77%). Between 2010 and 2018, the following complications showed the highest increase in rate per 1,000 hospitalized deliveries: blood transfusion with an increase of 1.49, acute renal failure with an increase of 0.20 and shock with an increase of 0.16. SMM indicator decreases occurred mainly for hysterectomy, disseminated intravascular coagulation, and ventilation. All the rate differences between the beginning and the end of the study period are described in Table 1.

**Table 1:** Delivery episodes with Severe Maternal Morbidity (SMM) Indicators and Rates of SMM Indicators per 1,000 Deliveries in Portugal by Year, from 2010 to 2018.

Indicator	2010		2011		2012		2013		2014		2015		2016		2017		2018		rate difference between 2018 vs. 2010	p*
	*n*	rate (95% CI)	*n*	rate (95% CI)	*n*	rate (95% CI)	*n*	rate (95% CI)	*n*	rate (95% CI)	*n*	rate (95% CI)	*n*	rate (95% CI)	*n*	rate (95% CI)	*n*	rate (95% CI)		
At least one Indicator of SMM	1029	11.89 (11.17–12.64)	1015	12.22 (11.48–12.99)	872	11.33 (10.59–12.11)	937	13.45 (12.6–14.34.6.34)	960	14.19 (13.31–15.12)	979	13.92 (13.06–14.82)	1042	14.02 (13.19–14.90)	973	13.11 (12.3–13.96.3.96)	1047	14.70 (13.82 15.62)	2.81	**0.048**
At least one Indicator of SMM, excluding blood transfusion	265	3.06 (2.70–3.45)	226	2.72 (2.38–3.10)	199	2.59 (2.24–2.97)	227	3.26 (2.85–3.71)	247	3.65 (3.21–4.14)	268	3.81 (3.37–4.29)	269	3.62 (3.20–4.08)	239	3.22 (2.83–3.66)	229	3.22 (2.81–3.66)	0.15	0.466
Acute Myocardial Infarction	2	0.02 (0.00- 0.08)	0	0.00 (0.00- 0.04)	0	0.00 (0.00–0.05.00.05)	0	0.00 (0.00–0.05.00.05)	1	0.01 (0.00- 0.08)	0	0.00 (0.00–0.05.00.05)	2	0.03 (0.00- 0.10)	1	0.01 (0.00–0.08.00.08)	0	0.00 (0.00–0.05.00.05)	−0.02	1.000
Aneurysm	0	0.00 (0.00- 0.04)	2	0.02 (0.00- 0.09)	1	0.01 (0.00- 0.07)	2	0.03 (0.00–0.10.00.10)	0	0.00 (0.00- 0.05)	1	0.01 (0.00- 0.08)	2	0.03 (0.00–0.10.00.10)	1	0.01 (0.00- 0.08)	0	0.00 (0.00- 0.05)	0.00	1.000
Acute Renal Failure	2	0.02 (0.00–0.08.00.08)	5	0.06 (0.02–0.14)	6	0.08 (0.03–0.17)	11	0.16 (0.08–0.28)	11	0.16 (0.08–0.29)	7	0.10 (0.04–0.21)	15	0.20 (0.11–0.33)	10	0.13 (0.06–0.25)	16	0.22 (0.13–0.36)	0.20	**0.009**
Acute Respiratory Distress Syndrome	20	0.23 (0.14–0.36)	24	0.29 (0.19–0.43)	23	0.30 (0.19–0.45)	29	0.42 (0.28–0.60)	24	0.35 (0.23–0.53)	22	0.31 (0.20–0.47)	21	0.28 (0.17–0.43)	17	0.23 (0.13–0.37)	14	0.20 (0.11–0.33)	−0.03	0.348
Amniotic Fluid Embolism	1	0.01 (0.00- 0.06)	3	0.04 (0.01–0.11)	3	0.04 (0.01–0.11)	2	0.03 (0.00–0.10.00.10)	2	0.03 (0.00–0.11.00.11)	1	0.01 (0.00–0.08.00.08)	0	0.00 (0.00–0.05.00.05)	1	0.01 (0.00–0.08.00.08)	1	0.01 (0.00–0.08.00.08)	0.00	0.252
Cardiac Arrest/Ventricular Fibrillation	2	0.02 (0.00–0.08.00.08)	1	0.01 (0.00–0.07.00.07)	2	0.03 (0.00- 0.09)	2	0.03 (0.00 0.10)	1	0.01 (0.00- 0.08)	1	0.01 (0.00–0.08.00.08)	4	0.05 (0.01–0.14)	0	0.00 (0.00- 0.05)	4	0.06 (0.02–0.14)	0.03	0.602
Conversion of Cardiac Rhythm	8	0.09 (0.04–0.18)	1	0.01 (0.00–0.07.00.07)	1	0.01 (0.00–0.07.00.07)	5	0.07 (0.02–0.17)	2	0.03 (0.00- 0.11)	1	0.01 (0.00- 0.08)	6	0.08 (0.03–0.18)	1	0.01 (0.00- 0.08)	4	0.06 (0.02–0.14)	−0.04	0.917
Disseminated Intravascular Coagulation	80	0.92 (0.73–1.15)	68	0.82 (0.64–1.04)	49	0.64 (0.47–0.84)	61	0.88 (0.67–1.12)	84	1.24 (0.99–1.54)	79	1.12 (0.89–1.4)	79	1.06 (0.84–1.33)	65	0.88 (0.68–1.12)	45	0.63 (0.46–0.85)	−0.29	0.917
Blood Transfusion	872	10.08 (9.42 10.77)	958	11.53 (10.81–12.29)	752	9.77 (9.09–10.5)	793	11.38 (10.61–12.21)	816	12.06 (11.25–12.92)	794	11.29 (10.51–12.10)	870	11.71 (10.94–12.51)	732	9.86 (9.16–10.61)	824	11.57 (10.79–12.39)	1.49	0.602
Eclampsia	30	0.35 (0.23–0.49)	22	0.26 (0.17–0.40)	24	0.31 (0.20–0.46)	14	0.20 (0.11–0.34)	13	0.19 (0.10–0.33)	23	0.33 (0.21–0.49)	21	0.28 (0.17–0.43)	19	0.26 (0.15–0.40)	14	0.20 (0.11–0.33)	−0.15	0.175
Heart Failure/Arrest During Surgery or Procedure	1	0.01 (0.00- 0.06)	0	0.00 (0.00–0.04.00.04)	1	0.01 (0.00- 0.07)	0	0.00 (0.00- 0.05)	1	0.01 (0.00- 0.08)	0	0.00 (0.00- 0.05)	0	0.00 (0.00- 0.05)	0	0.00 (0.00- 0.05)	0	0.00 (0.00–0.05.00.05)	−0.01	0.316
Puerperal Cerebrovascular Disorders	16	0.18 (0.11–0.30)	17	0.20 (0.12–0.33)	27	0.35 (0.23–0.51)	20	0.29 (0.18–0.44)	22	0.33 (0.20–0.49)	22	0.31 (0.20–0.47)	16	0.22 (0.12–0.35)	13	0.18 (0.09–0.30)	16	0.22 (0.13–0.36)	0.04	0.917
Pulmonary Edema/Acute Heart Failure	10	0.12 (0.06–0.21)	8	0.10 (0.04–0.19)	11	0.14 (0.07–0.26)	17	0.24 (0.14–0.39)	14	0.21 (0.11–0.35)	11	0.16 (0.08–0.28)	14	0.19 (0.10–0.32)	11	0.15 (0.07–0.27)	12	0.17 (0.09–0.29)	0.05	0.348
Severe Anesthesia Complications	27	0.31 (0.21–0.45)	12	0.14 (0.07–0.25)	21	0.27 (0.17–0.42)	18	0.26 (0.15–0.41)	25	0.37 (0.24–0.55)	41	0.58 (0.42–0.79)	28	0.38 (0.25–0.54)	21	0.28 (0.18–0.43)	28	0.39 (0.26–0.57)	0.08	0.118
Sepsis	18	0.21 (0.12–0.33)	12	0.14 (0.07–0.25)	13	0.17 (0.09–0.29)	12	0.17 (0.09–0.30)	11	0.16 (0.08–0.29)	14	0.20 (0.11–0.33)	18	0.24 (0.14–0.38)	9	0.12 (0.06–0.23)	12	0.17 (0.09–0.29)	−0.04	0.917
Shock	37	0.43 (0.30–0.59)	28	0.34 (0.22–0.49)	26	0.34 (0.22–0.50)	36	0.52 (0.36–0.72)	50	0.74 (0.55–0.97)	26	0.37 (0.24–0.54)	48	0.65 (0.48–0.86)	52	0.70 (0.52–0.92)	42	0.59 (0.42–0.80)	0.16	0.118
Sickle Cell Disease with Crisis	4	0.05 (0.01–0.12)	4	0.05 (0.01–0.12)	2	0.03 (0.00- 0.09)	6	0.09 (0.03–0.19)	1	0.01 (0.00–0.08.00.08)	4	0.06 (0.02–0.15)	5	0.07 (0.02–0.16)	4	0.05 (0.01–0.14)	3	0.04 (0.01–0.12)	0.00	0.917
Air and Thrombotic Embolism	15	0.17 (0.10–0.29)	7	0.08 (0.03–0.17)	6	0.08 (0.03–0.17)	4	0.06 (0.02–0.15)	8	0.12 (0.05–0.23)	6	0.09 (0.03–0.19)	15	0.20 (0.11–0.33)	5	0.07 (0.02–0.16)	8	0.11 (0.05–0.22)	−0.06	1.000
Hysterectomy	59	0.68 (0.52–0.88)	55	0.66 (0.50–0.86)	36	0.47 (0.33–0.65)	39	0.56 (0.40–0.77)	49	0.72 (0.54–0.96)	45	0.64 (0.47–0.86)	48	0.65 (0.48–0.86)	28	0.38 (0.25–0.55)	28	0.39 (0.26–0.57)	−0.29	0.175
Temporary Tracheostomy	0	0.00 (0.00–0.04.00.04)	1	0.01 (0.00- 0.07)	2	0.03 (0.00- 0.09)	0	0.00 (0.00–0.05.00.05)	1	0.01 (0.00–0.08.00.08)	1	0.01 (0.00- 0.08)	1	0.01 (0.00- 0.07)	1	0.01 (0.00- 0.08)	0	0.00 (0.00–0.05.00.05)	0.00	1.000
Ventilation	34	0.39 (0.27–0.55)	24	0.29 (0.19–0.43)	34	0.44 (0.31–0.62)	38	0.55 (0.39–0.75)	34	0.50 (0.35–0.70)	28	0.40 (0.26–0.58)	35	0.47 (0.33–0.66)	16	0.22 (0.12–0.35)	16	0.22 (0.13–0.36)	−0.17	0.602

CI: confidence interval; SMM: severe maternal morbidity.

*Mann-Kendall trend test.

Note: significant results (*p* < 0.05) in bold numerals.

The rate of SMM increased by 2.81 between 2010 and 2018. SMM, without including blood transfusion, increased by 0.15.

**Fig. 2:**
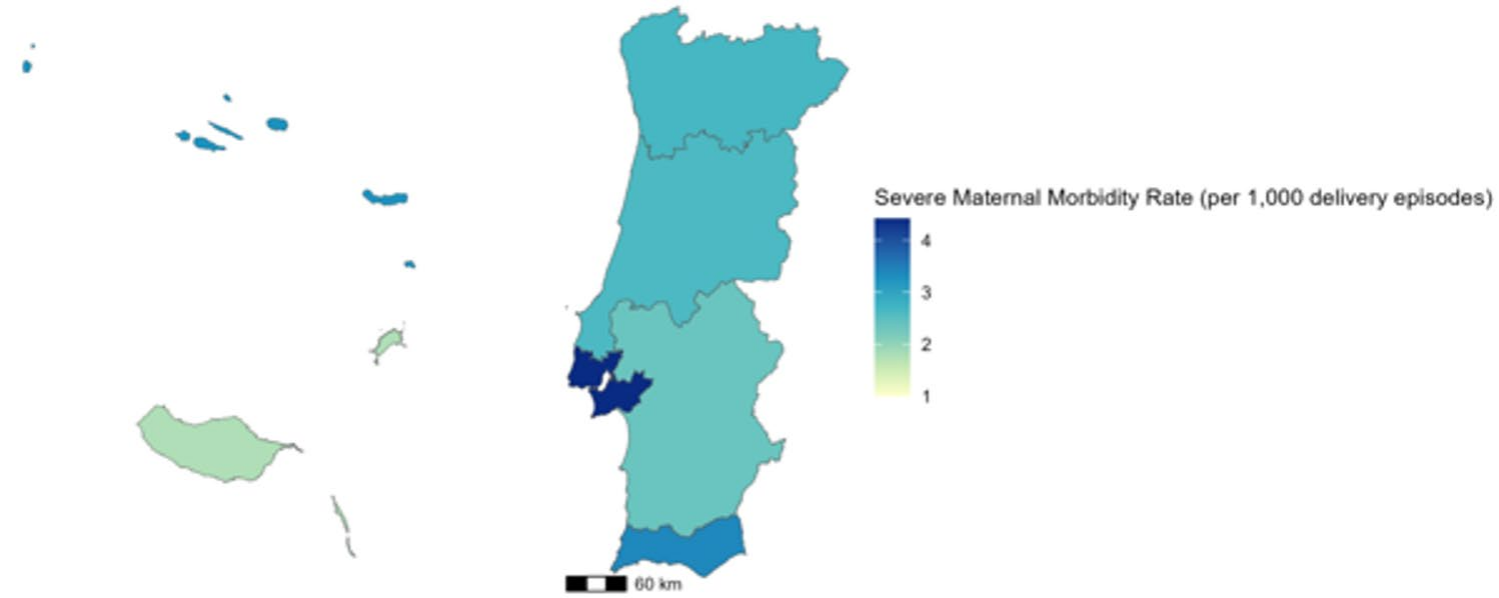
Data on Severe Maternal Morbidity (SMM) per 1,000 Deliveries per Nomenclature of Territorial Units for Statistics (NUTS) level II in Portugal.

SMM (excluding blood transfusion indicator) was numerically higher among women aged over 40 years, followed by women aged 35–39 years and 30–34 years, with rates of 7.4, 4.0 and 3.1 per 1,000 delivery episodes, respectively. Among younger groups, the rates were 2.8 per 1,000 for women under 20 years, 1.8 per 1,000 for those aged 20–24, and 2.7 per 1,000 for those aged 25–29. The geographical distribution according to NUTS level II, representing Portugal (Fig. 2), shows that the regions with the highest SMM rates were Lisbon Metropolitan Area (4.42 per 1,000 delivery episodes), followed by Algarve (3.38 per 1,000 delivery episodes), whereas the regions with the lowest rates were Madeira (1.77 per 1,000 delivery episodes) and Alentejo (2.37 per 1,000 delivery episodes).

Table 2 provides unadjusted and adjusted ORs to determine the association between demographic, clinical characteristics and ICD version with SMM occurrence; results for SMM with blood transfusion are listed in Supplementary Table S3. The logistic regression models demonstrated good discriminative ability, with an AUC of 0.821 and 0.812 for SMM without and with the blood transfusion indicator. Demographic and clinical characteristics were found to be associated with SMM in the unadjusted analysis. In the adjusted model, maternal age was significantly associated with the occurrence of SMM, particularly among women aged 40 years and above (adjusted OR 1.38, 95% CI 1.09–1.66). Hospitalized days had an adjusted OR of 1.04 (95% CI: 1.04–1.05), with each additional day of hospitalization representing a 4.4% increase in the odds of having SMM. The number of diagnoses (adjusted OR 1.27, 95% CI 1.25–1.28) and procedures (adjusted OR 1.26, 95% CI 1.24–1.27) recorded during hospitalization was associated with an increased odds for SMM. After 2015, a decrease in the odds of SMM was observed, from an adjusted OR of 0.75 (95% CI 0.57-0.57. 93) in 2015 to an adjusted OR of 0.36 (95% CI 0.22–0.95) in 2018. An increase in the OR for SMM was also shown for urgent admission (adjusted OR 1.04, 95% CI: 0.89–1.19) compared with planned admission, although not statistically significant. Compared with Lisbon Metropolitan Area, residing in Alentejo (adjusted OR 1.16, 95% CI: 0.96–1.37), Algarve (adjusted OR 1.21, 95% CI: 1.03–1.43) and Azores (adjusted OR 1.12, 95% CI: 0.64–1.60) showed an increase in the odds of SMM; whereas residing in the Norte and in Centro regions was associated with a decreased in the odds of SMM (adjusted OR 0.75, 95% CI: 0.64–0.86 and adjusted OR 0.82, 95% CI : 0.69–0.95, respectively). The transition to ICD-10-CM/PCS was also associated with increased odds of SMM (adjusted OR 1.63, 95% CI: 1.08–2.18). The direction and magnitude of results from sensitivity analyses using mixed effects logistic regression model remained consistent with the main findings (Supplementary Table S4).

In the analysis of SMM with the blood transfusion indicator (Supplementary Table S3), an increased SMM was significantly associated with days hospitalized (adjusted OR 1.04, 95% CI: 1.04–1.05), number of diagnoses (adjusted OR 1.33, 95% CI: 1.32–1.34) and procedures performed during hospitalization (adjusted OR 1.17, 95% CI: 1.16–1.17), and residing in the Algarve (adjusted OR 2.05 95% CI: 1.96–2.14), Alentejo (adjusted OR 1.29 95% CI: 1.19–1.39) or Centro (adjusted OR 1.28 95% CI: 1.22–1.34) regions. The transition to ICD-10-CM/PCS was also associated with increased odds of SMM (adjusted OR 1.42, 95% CI: 1.11–1.73). In contrast to the SMM without blood transfusion model, the SMM with blood transfusion indicator model showed that age was significantly associated with a decrease in SMM.

**Table 2:** Unadjusted and adjusted logistic regression for Severe Maternal Morbidity (SMM) without blood transfusions according to demographic and clinical variables.

Variable	Unadjusted OR (95% CI)		Adjusted OR (95% CI)	
Age group,				
< 20 years	Ref		Ref	
20–24 years	**0.677**	0.359–0.959	**0.707**	0.416–0.998
25–29 years	1.013	0.760–1.266	0.987	0.725–1.248
30–34 years	1.169	0.922–1.417	1.050	0.794–1.306
35–39 years	**1.577**	1.327–1.828	1.057	0.796–1.318
≥ 40 years	**2.617**	2.345–2.889	**1.375**	1.089–1.662
Hospitalization, days	**1.074**	1.071–1.078	**1.044**	1.040–1.047
Number of diagnostics	**1.417**	1.403–1.432	**1.268**	1.254–1.282
Number of procedures	**1.459**	1.440–1.477	**1.257**	1.243–1.272
NUTS level II				
Lisbon Metropolitan Area	Ref		Ref	
Norte	**0.591**	0.532–0.657	**0.754**	0.644–0.864
Alentejo	**0.487**	0.398–0.590	1.163	0.957–1.368
Algarve	**.0.678**	0.558–0.816	**1.213**	1.033–1.429
Centro	**0.552**	0.489–0.623	**0.821**	0.693–0.949
Madeira	**0.285**	0.172–0.440	0.705	0.207–1.204
Azores	0.655	0.395–1.012	1.122	0.640–1.604
Year				
2010	Ref		Ref	
2011	0.900	0.755–1.073	0.840	0.659–1.020
2012	0.855	0.709–1.029	**0.704**	0.512–0.896
2013	1.113	0.930–1.331	0.897	0.713–1.081
2014	**1.261**	1.058–1.502	0.904	0.723–1.084
2015	**1.294**	1.089–1.539	**0.746**	0.567–0.926
2016	**1.260**	1.061–1.495	**0.681**	0.500–0.862
2017	1.136	0.953–1.354	**0.401**	0.178–0.981
2018	0.717	0.623–1.359	**0.363**	0.219–0.945
Admission Type				
Planned	Ref		Ref	
Urgent	**0.717**	0.623–0.830	1.040	0.887–1.193
ICD Version				
ICD-9-CM	Ref		Ref	
ICD-10-CM/PCS	1.056	0.954–1.167	**1.629**	1.076–2.182

CI: confidence interval; ICD-09-CM: International Classification of Diseases Ninth Revision Clinical Modification; ICD-10-CM/PCS: International Classification of Diseases Tenth revision, Clinical Modification and Procedure Coding System; NUTS: Nomenclature of Territorial Units for Statistics; OR: odds ratio.

Note: significant results (*p* < 0.05) in bold numerals.

## Discussion

Due to the rarity of maternal mortality, the identification of women who experience severe adverse events during their hospitalization for delivery has been used to expand maternal care surveillance^17^. In this study, a population-level approach was used to assess the trends in SMM among delivery episodes in the Portuguese National Hospital Discharge Database from 2010 to 2018. The SMM rate for the study period was 13.14 per 1,000 deliveries, with a rate difference of 2.81 from 2010 to 2018, with the largest increase occurring in the blood transfusion indicator. Blood transfusion is considered an indicator of SMM and is part of the WHO’s severe acute maternal morbidity classification, also known as the “near miss”^18^, acting as a marker of critical clinical severity. It usually indicates a major obstetric complication where the patient experiences substantial blood loss or a life-threatening condition that requires urgent intervention to prevent maternal death.

Although our national SMM rates were numerically lower compared to other studies using the same SMM indicators^19–21^, we further excluded the blood transfusion indicator when assessing SMM to prevent the inclusion of false-positive cases^6,22^, as the codes used for transfusion do not specify the number of transfused units. In our analysis, when the blood transfusion indicator was not included, the SMM rate was 3.22 per 1,000 deliveries. In line with our findings, previous studies^19,21,23–26^have reported blood transfusion as the most common indicator of SMM. Fingar et al^25^. found that the increase in the SMM rate involving blood transfusion from 2006 to 2015 was over twice the increase in the rate of deliveries involving all the other indicators (54% versus 24% cumulative increase). This increase could be consistent with an increase in postpartum hemorrhage, which has been reported in several countries^27–30^. Following blood transfusion, disseminated intravascular coagulation and hysterectomy indicators were the most common indicators in women meeting SMM criteria, consistent with other studies^25,31^.

Recent changes in maternal characteristics, including increased age and pre-existing comorbidities^32^, have raised new concerns about SMM trends^33^ The higher documented rates of SMM in older mothers (> 35 years old) observed previously^19,32,34,35^ align with the findings from SMM without blood transfusions model. However, the effect of age was removed when considering the SMM model with blood transfusion. This may be because blood transfusion is often associated with specific complications, such as severe obstetric hemorrhage, that may not be directly influenced by maternal age^36^, and the transfusion indicator may act as a mediator or confounder, absorbing some of the variance in SMM that would otherwise be attributed to age.

Efforts to enhance maternal care at a national level, including the training of healthcare professionals and the reorganization of maternal and perinatal health systems, likely contributed to reducing the risk of SMM after 2015. However, our findings suggest geographical inequalities in access to maternal and perinatal care. These may be partly explained by the uneven distribution of maternity units, with a concentration in coastal and urban areas of Portugal^37^. The 2006 restructuring of the maternal healthcare system^38^, while aimed at improving safety and quality, may have inadvertently introduced spatial inequalities in access to timely and specialized obstetric care, especially in rural and inland regions. Additionally, higher SMM rates in urban centers may reflect the centralization of high-risk pregnancies in referral hospitals, which manage more complex cases. However, these analyses were not designed to formally assess spatial patterns or healthcare accessibility. Although our sensitivity analysis accounting for hospital-level clustering remained consistent with the main results, future research should incorporate more detailed data, including municipality- and hospital-level data, to better characterize geographic inequalities and access to obstetric care.

Consistent with our findings, Guglielminotti et al^39^. suggested that a higher coding intensity, as reflected by the number of diagnosis and procedure codes, was associated with an increased risk of SMM. Our results also showed an association between the length of hospitalization and SMM. It is important to note that the number of diagnoses, procedures, and length of hospitalization are associated with greater clinical complexity in women already experiencing SMM, indicating the increased medical attention and interventions needed. These associations likely reflect the reverse causality link between women with SMM and higher numbers of complications, the need for procedures, and the requirement for longer hospital care, rather than acting as independent risk factors.

An association was also found between the transition to the ICD-10-CM/PCS version and SMM. One of the improved features of the ICD-10-CM/PCS classification system is the increased granularity and specificity in categorizing health conditions^15^. For example, in ICD-9-CM, 10 codes were available to code blood transfusion as a procedure, whereas ICD-10-CM/PCS has 64 codes. Therefore, the rise in SMM rates, especially for blood transfusions, should be viewed cautiously, as it could be partly due to the coding system transition rather than a real change in maternal health.

Strengths of this study include an epidemiological overview of SMM indicators from a large national database, providing insights into the Portuguese context of maternal care. Nevertheless, our study results should consider the following limitations. Limited availability of data from hospitals in the autonomous regions of Portugal, namely the islands of Madeira and the Azores, potentially under-representing these regions^40^. Data from private hospitals, which account for 19.6% of Portugal’s total delivery episodes^13^, were not included. This limits the generalizability of the results outside the public Portuguese healthcare system and may lead to biased SMM estimates. Women delivering in private hospitals may have different risk profiles, such as higher socioeconomic status^41^ and fewer comorbidities^42^, compared to those in public hospitals. As a result, our findings might overestimate SMM rates if lower-risk women tend to deliver in private hospitals.

Although the SMM developed by the CDC is a validated method, there is still likely a degree of misclassification^22^. A certain level of errors or misclassification are also expected since the data for SMM and its indicators are recorded during routine clinical practice and coded for administrative purposes, not specifically for this research. The increase in the blood transfusion indicator observed in our study requires careful interpretation. This may not only indicate a true rise in obstetric hemorrhage but could also be due to changes in clinical practices, such as more liberal transfusion thresholds or new guidelines. The transition from ICD-9-CM/PCS to ICD-10-CM/PCS, with its substantially greater granularity for transfusion codes, likely influenced the observed trend. Furthermore, blood transfusion is recognized as a less specific indicator of SMM. These limitations highlight the need for cautious interpretation of SMM rates and underscore the importance of considering both clinical and coding factors when evaluating maternal health trends. Reverse causality is also a limitation of the current study design. The use of administrative hospital discharge data and code-based definitions of SMM introduces tautological associations, as it is reasonable to expect that SMM may result in higher levels of subsequent complications, represented by the intensity of diagnosis and procedure coding, and the requirement for longer hospital care. It is also important to consider the potential for coding bias, where hospitals with more comprehensive or systematic documentation practices may record a higher number of diagnoses and procedures, which could artificially inflate SMM rates in administrative data. Due to the limited extent of the database it was not possible to determine whether the conditions that classified the SMM occurred during delivery or postpartum hospitalization, nor the severity of these conditions. In addition, this analysis did not take into account other relevant factors, such as race, lifestyle and socioeconomic factors, presence of comorbidities, and prenatal care, and despite adjustment for available demographic and clinical variables, there remains a risk of residual confounding from unmeasured factors. Future research should employ longitudinal designs to better establish the temporal sequence of events and minimize bias. It should also incorporate a more comprehensive selection of potential risk factors.

In conclusion, an overview of SMM trends in hospital admission episodes with a diagnosis related to delivery between 2010 and 2018 in Portugal was presented. In our findings, trends in deliveries with SMM show an increase, particularly for blood transfusion, acute renal failure and shock. Risk factors associated with SMM included age, length of hospitalization, intensity of diagnosis and procedure coding, and the transition to ICD-10-CM/PCS coding. To our knowledge, this is the first study to examine SMM rates at the national level, which can provide us with a landscape of maternal care and serve as a basis for future monitoring activities in obstetric care and management.

## Supplementary Information

Below is the link to the electronic supplementary material.

Supplementary Material 1

## Data Availability

The data supporting the findings of this study are not publicly available. They were obtained under a protocol established between the Central Administration of the Health System (ACSS) and the Faculty of Medicine of the University of Lisbon (FMUL) for scientific research purposes. Due to legal and ethical restrictions, these data cannot be shared with third parties.
