# Supplementary material for: Trends and factors associated with delivery hospitalizations involving severe maternal morbidity in portuguese public hospitals: A population-based study (2010–2018)
Source: Sci Rep. 2026 Apr 4;16:16232. doi: 10.1038/s41598-026-42363-9 (PMC13201652; doi:10.1038/s41598-026-42363-9)
Supplement: Supplementary file 1 — Supplementary Material 1 [file 41598_2026_42363_MOESM1_ESM.docx]

Table S1. Delivery identification elements and identification codes.

| **Description of the elements** | **ICD-9-CM Code(s)** | **ICD-10-CM/PCS Code(s)** |
| --- | --- | --- |
| Outcome of delivery | V27.0 Outcome of delivery, single liveborn  V27.1 Outcome of delivery, single stillborn  V27.2 Outcome of delivery, twins, both liveborn  V27.3 Outcome of delivery, twins, one liveborn and one stillborn  V27.4 Outcome of delivery, twins, both stillborn  V27.5 Outcome of delivery, other multiple birth, all liveborn  V27.6 Outcome of delivery, other multiple birth, some liveborn  V27.7 Outcome of delivery, other multiple birth, all stillborn  V27.9 Outcome of delivery, unspecified outcome of delivery | Z37.0 Single live birth  Z37.1 Single stillbirth  Z37.2 Twins, both liveborn  Z37.3 Twins, one liveborn and one stillborn  Z37.4 Twins, both stillborn  Z37.50 Multiple births, unspecified, all liveborn  Z37.51 Triplets, all liveborn  Z37.52 Quadruplets, all liveborn  Z37.53 Quintuplets, all liveborn  Z37.54 Sextuplets, all liveborn  Z37.59 Other multiple births, all liveborn  Z37.60 Multiple births, unspecified, some liveborn  Z37.61 Triplets, some liveborn  Z37.62 Quadruplets, some liveborn  Z37.63 Quintuplets, some liveborn  Z37.64 Sextuplets, some liveborn  Z37.69 Other multiple births, some liveborn  Z37.7 Other multiple births, all stillborn  Z37.9 Outcome of delivery, unspecified |
| Normal delivery | 650 Normal delivery | O80 Encounter for full-term uncomplicated delivery  O82 Encounter for cesarean delivery without indication |
| Diagnosis-related group (DRG) delivery codes | 370 Complicated cesarean section  371 Uncomplicated cesarean section  372 Complicated vaginal delivery  373 Uncomplicated vaginal delivery  374 Uncomplicated vaginal delivery with sterilization and/or dilatation & curettage  375 Vaginal delivery with operation room procedure except sterilization and/or dilatation & curettage | 765 Complicated cesarean section  766 Uncomplicated cesarean section  767 Uncomplicated vaginal delivery with sterilization and/or dilation and curettage  768 Vaginal delivery with operation room procedure except sterilization and/or dilation and curettage  774 Complicated vaginal delivery  775 Uncomplicated vaginal delivery |
| Selected delivery related procedures | 72.0 Low forceps operation  72.1 Low forceps operation with episiotomy  72.21 Mid forceps operation with episiotomy  72.29 Other mid forceps operation  72.31 High forceps operation with episiotomy  72.39 Other high forceps operation  72.4 Forceps rotation of fetal head  72.51 Partial breech extraction with forceps to aftercoming head  72.52 Other partial breech extraction  72.53 Total breech extraction with forceps to aftercoming head  72.54 Other total breech extraction  72.6 Forceps application to aftercoming head  72.71 Vacuum extraction with episiotomy  72.79 Other vacuum extraction  72.8 Other specified instrumental delivery  72.9 Unspecified instrumental delivery  73.22 Internal and combined version with extraction  73.59 Other manually assisted delivery  73.6 Episiotomy  74.0 Classical cesarean section  74.1 Low cervical cesarean section  74.2 Extraperitoneal cesarean section  74.4 Cesarean section of other specified type  74.99 Other cesarean section of unspecified type | 10D00Z0 Extraction of Products of Conception, High, Open Approach  10D00Z1 Extraction of Products of Conception, Low, Open Approach  10D00Z2 Extraction of Products of Conception, Extraperitoneal, Open Approach  10D07Z3 Extraction of Products of Conception, Low Forceps, Via Natural or Artificial Opening  10D07Z4 Extraction of Products of Conception, Mid Forceps, Via Natural or Artificial Opening  10D07Z5 Extraction of Products of Conception, High Forceps, Via Natural or Artificial Opening  10D07Z6 Extraction of Products of Conception, Vacuum, Via Natural or Artificial Opening  10D07Z7 Extraction of Products of Conception, Internal Version, Via Natural or Artificial Opening  10D07Z8 Extraction of Products of Conception, Other, Via Natural or Artificial Opening  10E0XZZ Delivery of Products of Conception, External Approach |
| ICD-09-CM: International Classification of Diseases Ninth Revision Clinical Modification; ICD-10-CM/PCS: International Classification of Diseases Tenth edition, Clinical Modification/Procedure Coding System. | | |

Table S2. Severe Maternal Morbidity (SMM) indicators and corresponding identification codes.

| **Severe Maternal Morbidity Indicator** | **Diagnosis (DX) or Procedure (PR) Code** | **ICD-9** | **ICD-10** |
| --- | --- | --- | --- |
| **Acute Myocardial Infarction** | DX | 410.xx | I21.xx, I22.x |
| **Aneurysm** | DX | 441.xx | I71.xx, I79.0 |
| **Acute Renal Failure** | DX | 584.5, 584.6, 584.7, 584.8, 584.9, 669.3x | N17.x, O90.4 |
| **Acute Respiratory Distress Syndrome** | DX | 518.5x, 518.81, 518.82, 518.84, 799.1 | J80, J95.1, J95.2, J95.3, J95.82x, J96.0x, J96.2x, J96.9x, R06.03, R09.2 |
| **Amniotic Fluid Embolism** | DX | 673.1x | O88.112, O88.113, O88.119, O88.12, O88.13 |
| **Cardiac Arrest / Ventricular Fibrillation** | DX | 427.41, 427.42, 427.5 | I46.x, I49.0x |
| **Conversion of Cardiac Rhythm** | PR | 99.6x | 5A12012, 5A2204Z |
| **Disseminated Intravascular Coagulation** | DX | 286.6, 286.9, 641.3x, 666.3x | D65, D68.8, D68.9, O45.002, O45.003, O45.009, O45.012, O45.013, O45.019, O45.022, O45.023, O45.029, O45.092, O45.093, O45.099, O46.002, O46.003, O46.009, O46.012, O46.013, O46.019, O46.022, O46.023, O46.029, O46.092, O46.093, O46.099, O67.0, O72.3 |
| **Blood Transfusion** | PR | 99.0x | 30230H0, 30230K0, 30230L0, 30230M0, 30230N0, 30230P0, 30230R0, 30230T0, 30230H1, 30230K1, 30230L1, 30230M1, 30230N1, 30230P1, 30230R1, 30230T1, 30233H0, 30233K0, 30233L0, 30233M0, 30233N0, 30233P0, 30233R0, 30233T0, 30233H1, 30233K1, 30233L1, 30233M1, 30233N1, 30233P1, 30233R1, 30233T1, 30240H0, 30240K0, 30240L0, 30240M0, 30240N0, 30240P0, 30240R0, 30240T0, 30240H1, 30240K1, 30240L1, 30240M1, 30240N1, 30240P1, 30240R1, 30240T1, 30243H0, 30243K0, 30243L0, 30243M0, 30243N0, 30243P0, 30243R0, 30243T0, 30243H1, 30243K1, 30243L1, 30243M1, 30243N1, 30243P1, 30243R1, 30243T1 |
| **Eclampsia** | DX | 642.6x | O15. X |
| **Heart Failure / Arrest During Surgery or Procedure** | DX | 997.1 | I97.120, I97.121, I97.130, I97.131, I97.710, I97.711 |
| **Puerperal Cerebrovascular Disorders** | DX | 046.3, 348.39, 362.34, 430.xx, 431.xx, 432.xx, 433.xx, 434.xx, 435.xx, 436.xx, 437.xx, 671.5x, 674.0x, 997.02 | A81.2, G45.x, G46.x, G93.49, H34.0x, I60.xx, I61.xx, I62.xx, I63.00, I63.01x, I63.1xx, I63.2xx, I63.3xx, I63.4xx, I63.5xx, I63.6, I63.8x, I63.9, I65.xx, I66.xx, I67.xx, I68.xx, O22.50, O22.52, O22.53, I97.810, I97.811, I97.820, I97.821, O87.3 |
| **Pulmonary Edema / Acute Heart Failure** | DX | 428.0, 428.1, 428.20, 428.21, 428.23, 428.30, 428.31, 428.33, 428.40, 428.41, 428.43, 428.9, 518.4 | I50.1, I50.20, I50.21, I50.23, I50.30, I50.31, I50.33, I50.40, I50.41, I50.43, I50.810, I50.811, I50.813, I50.814, I50.82, I50.83, I50.84, I50.89, I50.9, J81.0 |
| **Severe Anesthesia Complications** | DX | 668.0x, 668.1x, 668.2x, 995.4, 995.86 | O29.112–O29.119, O29.122–O29.129, O29.192–O29.199, O29.212–O29.219, O29.292–O29.299, O74.0, O74.1, O74.2, O74.3, O89.0x, O89.1, O89.2, T88.2XXA, T88.3XXA |
| **Sepsis** | DX | 038.xx, 449, 785.52, 995.91, 995.92, 998.02, 670.2x (after October 1, 2009) | A32.7, A40.x, A41.x, I76, O85, O86.04, R65.20, R65.21, T81.12XA, T81.44XA |
| **Shock** | DX | 669.1x, 785.50, 785.51, 785.59, 995.0, 998.0*, 998.00, 998.01, 998.09 *998.0 is not a valid code but was used prior to 2012 | O75.1, R57.x, T78.2XXA, T81.10XA, T81.11XA, T81.19XA, T88.6XXA |
| **Sickle Cell Disease With Crisis** | DX | 282.42, 282.62, 282.64, 282.69, 289.52 | D57.00, D57.01, D57.02, D57.211, D57.212, D57.219, D57.411, D57.412, D57.419, D57.811, D57.812, D57.819 |
| **Air and Thrombotic Embolism** | DX | 415.0, 415.1x, 673.0x, 673.2x, 673.3x, 673.8x | I26.x, O88.012–O88.03, O88.212–O88.23, O88.312–O88.33, O88.812–O88.83, T80.0XXA |
| **Hysterectomy** | PR | 68.39, 68.49, 68.59, 68.69, 68.79, 68.9 (also include 68.3, 68.4, 68.5, 68.6, 68.7; non-specific codes used frequently) | 0UT90ZL, 0UT90ZZ, 0UT97ZL, 0UT97ZZ |
| **Temporary Tracheostomy** | PR | 31.1 | 0B110F4, 0B113F4, 0B114F4 |
| **Ventilation** | PR | 96.70, 96.71, 96.72 | 5A1935Z, 5A1945Z, 5A1955Z |
| DX: Diagnosis; ICD-09-CM: International Classification of Diseases Ninth Revision Clinical Modification; ICD-10-CM/PCS: International Classification of Diseases Tenth Revision, Clinical Modification/Procedure Coding System; PR: Procedure | | | |

Table S3. Unadjusted and adjusted logistic regression for Severe Maternal Morbidity (SMM) with blood transfusion indicator according to demographic and clinical variables.

| **Variable** | **Unadjusted OR (95% CI)** | | **Adjusted OR (95% CI)** | |
| --- | --- | --- | --- | --- |
| Age groups |  |  |  |  |
| <20 years | **Ref** |  | **Ref** |  |
| 20 – 24 years | **0.723** | 0.610 - 0.836 | **0.735** | 0.618 - 0.852 |
| 25 – 29 years | **0.707** | 0.603 - 0.812 | **0.664** | 0.555 - 0.774 |
| 30 – 34 years | **0.761** | 0.659 - 0.864 | **0.654** | 0.547 - 0.761 |
| 35 – 39 years | **0.897** | 0.791 - 1.002 | **0.561** | 0.450 - 0.672 |
| ≥ 40 years | **1.225** | 1.101 - 1.350 | **0.612** | 0.479 - 0.745 |
| Hospitalization, days | **1.073** | 1.071 - 1.076 | **1.042** | 1.039 - 1.045 |
| Number of diagnostics | **1.388** | 1.380 - 1.396 | **1.331** | 1.323 - 1.338 |
| Number of procedures | **1.334** | 1.325 - 1.343 | **1.166** | 1.159 - 1.174 |
| NUTS level II |  |  |  |  |
| Lisbon | Ref |  | Ref |  |
| Norte | **0.660** | 0.626 - 0.697 | **0.834** | 0.778 - 0.891 |
| Alentejo | **0.569** | 0.516 - 0.626 | **1.288** | 1.187 - 1.389 |
| Algarve | 1.063 | 0.977 - 1.155 | **2.050** | 1.962 - 2. 138 |
| Centro | **0.815** | 0.770 - 0.862 | **1.282** | 1.222 - 1.342 |
| Madeira | **0.135** | 0.093 - 0.189 | **0.340** | 0.024 - 0.703 |
| Azores | **0.494** | 0.367 - 0.648 | 0.766 | 0.473 - 1.058 |
| Year |  |  |  |  |
| 2010 | Ref |  | Ref |  |
| 2011 | 1.063 | 0.976 - 1.158 | 1.018 | 0.930 - 1.105 |
| 2012 | 0.962 | 0.878 - 1.053 | **0.852** | 0.758 - 0.946 |
| 2013 | **1.177** | 1.077 - 1.287 | 0.980 | 0.888 - 1.072 |
| 2014 | **1.264** | 1.156 - 1.381 | 0.963 | 0.871 - 1.054 |
| 2015 | **1.242** | 1.137 - 1.357 | **0.792** | 0.700 - 0.884 |
| 2016 | **1.254** | 1.150 - 1.368 | **0.767** | 0.675 - 0.858 |
| 2017 | **1.170** | 1.071 - 1.278 | **0.474** | 0.156 - 0.793 |
| 2018 | **1.315** | 1.206 - 1.433 | **0.521** | 0.202 - 0.840 |
| Admission Type |  |  |  |  |
| Planned | Ref |  | Ref |  |
| Urgent | **0.787** | 0.732 - 0.847 | 1.064 | 0.986 - 1.143 |
| ICD version |  |  |  |  |
| ICD-9-CM | Ref |  | Ref |  |
| ICD-10-CM/PCS | **1.094** | 1.041 - 1.149 | **1.419** | 1.113 - 1.725 |
| CI: confidence interval; ICD-09-CM: International Classification of Diseases Ninth Revision Clinical Modification; ICD-10-CM/PCS: International Classification of Diseases Tenth revision, Clinical Modification and procedure coding system; NUTS: Nomenclature of Territorial Units for Statistics; OR: odds ratio.  Note: significant results (p<0.05) in bold numerals. | | | | |

Table S4. Multilevel mixed-effects adjusted logistic regression model for Severe Maternal Morbidity (SMM) without blood transfusions indicator according to demographic and clinical variables with a random intercept for hospital

| **Variable** | **Adjusted OR** | **95% CI** | **p-value** |
| --- | --- | --- | --- |
| (Intercept) | 0.000 | 0.000 – 0.000 | <0.001 |
| Age, years |  |  |  |
| <20 years | **0.719** | 0.537 – 0.963 | **0.027** |
| 20 – 24 years | 1.026 | 0.789 – 1.335 | 0.849 |
| 25 – 29 years | 1.118 | 0.864 – 1.447 | 0.398 |
| 30 – 34 years | 1.103 | 0.848 – 1.434 | 0.466 |
| ≥40 years | **1.434** | 1.073 – 1.916 | **0.015** |
| Hospitalization, days | **1.032** | 1.028 – 1.036 | **<0.001** |
| Number of diagnostics | **1.301** | 1.280 – 1.322 | **<0.001** |
| Number of procedures | **1.391** | 1.367 – 1.415 | **<0.001** |
| NUTS level II |  |  |  |
| Lisbon Metropolitan Area | Ref |  |  |
| Norte | 0.726 | 0.490 – 1.076 | 0.111 |
| Alentejo | 1.167 | 0.843 – 1.617 | 0.352 |
| Algarve | 0.823 | 0.414 – 1.634 | 0.577 |
| Centro | 0.765 | 0.564 – 1.039 | 0.086 |
| Madeira | 0.492 | 0.158 – 1.533 | 0.221 |
| Azores | 0.733 | 0.361 – 1.489 | 0.390 |
| Year |  |  |  |
| 2010 | Ref |  |  |
| 2011 | 0.837 | 0.693 – 1.010 | 0.063 |
| 2012 | **0.768** | 0.626 – 0.943 | **0.012** |
| 2013 | 0.898 | 0.733 – 1.100 | 0.298 |
| 2014 | 0.870 | 0.711 – 1.064 | 0.174 |
| 2015 | **0.767** | 0.629 – 0.936 | **0.009** |
| 2016 | **0.683** | 0.559 – 0.836 | **<0.001** |
| 2017 | **0.458** | 0.250 – 0.836 | **0.011** |
| 2018 | **0.420** | 0.229 – 0.770 | **0.005** |
| Admission Type |  |  |  |
| Planned | Ref |  |  |
| Urgent | 1.000 | 0.852 – 1.173 | 0.998 |
| ICD Version |  |  |  |
| ICD-9 | Ref |  |  |
| ICD-10 | 1.615 | 0.912 – 2.861 | 0.100 |
| CI: confidence interval; ICC: Intraclass Correlation Coefficient; ICD-09-CM: International Classification of Diseases Ninth Revision Clinical Modification; ICD-10-CM/PCS: International Classification of Diseases Tenth revision, Clinical Modification and procedure coding system; NUTS: Nomenclature of Territorial Units for Statistics; OR: odds ratio.  Note: significant results (p<0.05) in bold numerals. | | | |
